# Intranasal Midazolam Prior to Enema for Management of Constipation in the Pediatric Emergency Department

**DOI:** 10.7759/cureus.82919

**Published:** 2025-04-24

**Authors:** Brian L Park, Emine Tunc, Pingping Qu, Eileen Klein, Patrick Solari

**Affiliations:** 1 Emergency Department, University of California San Diego, San Diego, USA; 2 Pediatric Emergency Medicine, University of Texas Southwestern Medical Center, Dallas, USA; 3 Biostatistics and Epidemiology, Seattle Children's Hospital, Seattle, USA; 4 Pediatric Emergency Medicine, Seattle Children's Hospital/University of Washington, Seattle, USA

**Keywords:** anxiolysis, constipation, emergency medicine, enema, pediatrics

## Abstract

Introduction: Constipation is a common complaint in the pediatric emergency department (PED). Management often includes an enema administered in the PED, especially for children with significant discomfort or those who have failed outpatient therapy with oral bowel motility agents. Enema administration can be anxiety-provoking and may cause children to further withhold stool. Midazolam, a rapid-acting benzodiazepine, is sometimes given prior to an enema for anxiolysis. This study evaluated the effect of intranasal midazolam on the success rate of bowel movement after an enema for the management of constipation in the pediatric emergency department (PED).

Methods: Retrospective cohort study at a single, academic, quaternary-care hospital. Patients 2 to 10 years of age receiving at least one enema for management of constipation from May 1, 2016, through April 30, 2021, were included. Exclusion criteria include neurodevelopmental disorders, prior abdominal surgeries, or administration of any non-intranasal midazolam anxiolytic agents. The primary outcome was the success of bowel movement in the emergency department (ED). Secondary outcomes included ED length-of-stay (LOS), enema-to-discharge time, number of enemas administered, and admission rates.

Results: Intranasal midazolam was administered in 214 (27%) out of 795 encounters. There was no difference in rates of successful bowel movements between the midazolam and no-midazolam groups (83.6% vs. 84.2%, respectively). When stratified by age in multivariate regression analysis, the rate of bowel movement success was higher in preschool-aged children receiving midazolam (ARR 1.09; CI 1.01-1.18, p=0.032). Midazolam was associated with longer ED LOS (271 min vs. 235 min, p=0.004), longer enema-to-discharge time (121 min vs. 93 min, p < 0.001), more frequent administration of multiple enemas (14% vs. 7%, p=0.021), and higher admission rates (7% vs. 2%, p=0.002).

Conclusions: Intranasal midazolam prior to enema administration was not associated with an overall higher rate of successful bowel movement in the PED. Pre-school-aged children showed a small improvement in bowel movement success with midazolam. Midazolam administration was associated with worse secondary outcomes, but there are likely other confounding factors such as severity of illness. Intranasal midazolam may provide benefits to preschool-aged children, but a prospective study is needed to confirm this finding.

## Introduction

Constipation is a common pediatric problem and accounts for more than 400,000 annual emergency department (ED) visits by children [1]. While the majority of children with constipation have no identifiable anatomic or metabolic pathology, it can be a significant source of pain for children and distress for their families [2]. An enema is an effective tool in the acute management of constipation in the ED, especially for children with stool impaction who have failed outpatient therapy with oral bowel motility agents [3]. However, an enema is often an anxiety-provoking procedure and can precipitate behavioral disturbances, including agitation and crying, which may prevent the child from cooperating with or tolerating enema administration by the ED staff. This leads to a poor experience for the child and family and may cause the child to further withhold stool.

Midazolam, a rapid-acting benzodiazepine, is frequently used to provide anxiolysis for common procedures in the ED, such as laceration repairs and foreign body removals. Midazolam is often administered intranasally (IN) via an atomizer due to rapid and predictive onset of action, not requiring intravenous (IV) access, and a well-established safety profile and clinical efficacy [4-9]. Another benefit of midazolam is the potential for antegrade amnesia, which may reduce the psychological trauma from a potentially stressful procedure in the ED [10]. For these reasons, some emergency physicians and nurse practitioners in our ED choose to administer IN midazolam prior to enema administration. In our group’s experience, children premedicated with IN midazolam anecdotally appear to tolerate the procedure better and have more rapid bowel movements (BM). Thus, we endeavored to study these observations.

The primary objective of the study was to evaluate the efficacy and safety of IN midazolam for anxiolysis prior to enema administration by comparing the clinical outcomes between children who were premedicated with IN midazolam and those who were not. We hypothesized that children premedicated with IN midazolam prior to an enema would have a higher rate of successful BM compared to those who were not premedicated with IN midazolam by providing anxiolysis and decreasing maladaptive behaviors such as intentional stool retention and agitation.

## Materials and methods

Study design

We conducted a retrospective cohort study at a single, academic, quaternary-care pediatric ED with more than 50,000 annual visits. This study was exempted by our institutional review board.

Patients 2 to 10 years of age receiving at least one enema for management of constipation in the pediatric ED from May 1, 2016, through April 30, 2021, were included in the study. This age range was chosen to include children with the highest prevalence of functional constipation (2 to 4 years of age) as well as older children for whom anxiolysis may be beneficial. Exclusion criteria included neurodevelopmental disorders, prior abdominal surgeries, administration of any anxiolytic other than IN midazolam prior to enema administration, and incomplete documentation of a successful BM. Neurodevelopmental disorders include any motor or cognitive impairments or sensory processing issues that may be associated with a higher prevalence of constipation, such as autism spectrum disorder and cerebral palsy [11,12]. Prior abdominal surgeries include any surgical instrumentation in the abdominal cavity, including, but not limited to, appendectomy, bowel resection, and gastrostomy tube placement. These exclusion criteria were selected to evaluate the effect of IN midazolam in neurotypical children with normal gastrointestinal function and anatomy.

All visits with any enema administration in our ED during the study period were extracted from our electronic medical records (EMR) (Cerner Corp., Kansas City, MO, USA; EPIC Systems Corp., Verona, WI, USA) by our institution’s data analytic service. Enemas included in the search were bisacodyl, mineral oil, and 0.9% sodium chloride. All eligible encounters were reviewed by two pediatric emergency medicine fellows for inclusion and exclusion criteria, patient demographics, clinical history and physical exam findings, clinical outcomes, and imaging studies. Specific clinical history of interest included the use of bowel motility agents prior to the ED encounter. This was defined as three or more consecutive days of an oral motility agent (e.g., polyethylene glycol 3350, sennosides, bisacodyl) to allow adequate time for these medications to take effect, or any enema administration within one day of the index ED visit. Some patients had multiple ED visits with enema administration. To avoid duplicating a single constipation episode, subsequent ED visits occurring within one week of the initial visit were excluded.

IN midazolam was dosed at 0.4 mg/kg and given through either nostril or divided between both nostrils depending on the volume of medication via MAD Nasal™Atomization Device (Teleflex Inc., Wayne, PA, USA), typically 5 to 10 minutes prior to enema administration. Enema options were bisacodyl 5-10 mg, sodium chloride 0.9% 15-20 mL/kg, or mineral oil 30-60ml. The decision to administer IN midazolam and the timing of enema administration after midazolam was based on physician discretion. Repeat doses of IN midazolam are generally not provided at our institution if the patient requires additional enemas to minimize the risk of adverse events.

The primary outcome was whether the patient had a BM during the ED visit after receiving at least one enema. A successful BM was defined by EMR documentation of a BM after receiving an enema. An unsuccessful BM was defined as no or minimal stooling before discharge from our ED, as documented in the EMR. Secondary outcomes included ED length of stay (LOS) in minutes, enema-to-discharge time in minutes, the proportion of patients who received more than one enema during the same ED visit (among patients who were not admitted to the hospital), admissions for constipation management, and adverse events due to IN midazolam. ED LOS and enema-to-discharge time were evaluated to account for the time required for midazolam preparation and administration. Given the study’s retrospective design and difficulty in assessing clinical severity, the number of enemas administered and admissions for constipation management were used as indirect markers of constipation severity and clinician concern. Adverse events included respiratory depression, bradycardia, hypotension, paradoxical reaction, vomiting, allergic or anaphylactic reaction, or death.

Statistical analysis

Demographic characteristics were summarized using descriptive statistics such as medians and interquartile ranges (IQR) for continuous variables and frequencies and percentages for categorical variables. An extension of the modified Poisson regression analysis [13] was used to examine the association between our binary outcomes and midazolam use while taking into account potential correlations among all outcome values within patients due to multiple visits. Covariates to adjust for included age, gender, ethnicity, and prior bowel motility use. This modeling approach allowed us to estimate the risk ratio (RR) and its 95% confidence interval (CI) for each binary outcome, comparing the patients who received IN Midazolam to those who did not. The analysis was implemented using generalized estimation equations (GEE) with a Poisson link and exchangeable correlation structure with the R geepack package [14]. For the primary outcome of whether the patient had a BM in the ED, stratified analyses were also conducted in each age group: toddler (<3 years), preschool (3 to <6 years), and school-aged (6 to 10 years). For the continuous outcomes (ED LOS in minutes and enema-to-discharge time in minutes), GEE was employed with a Gaussian link in conjunction with an exchangeable correlation structure.

## Results

**Figure 1:**
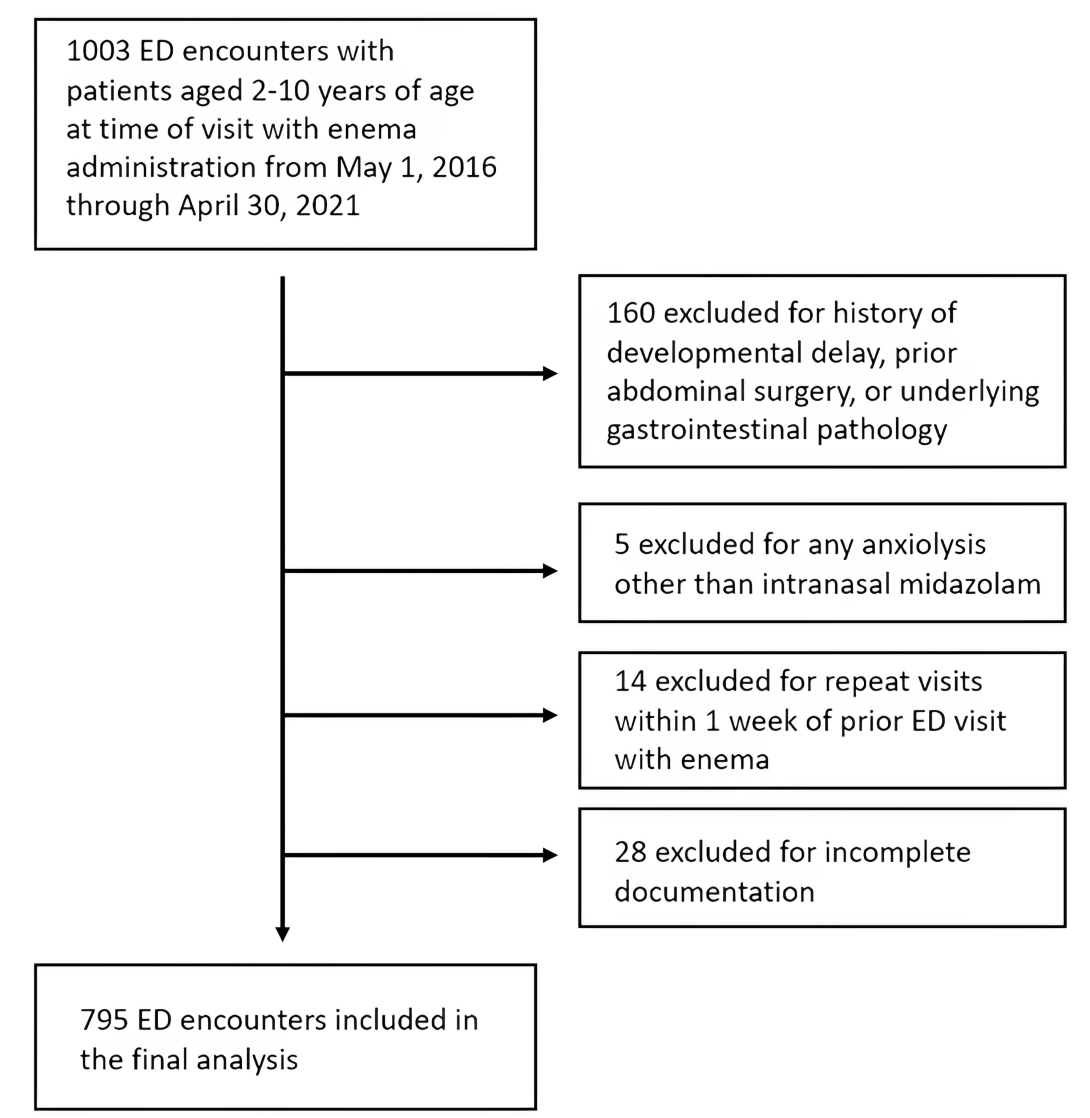
Study inclusion and exclusion flowchart

A total of 795 unique ED encounters met the inclusion and exclusion criteria out of the 1005 encounters with enema administration in our ED for management of constipation from May 1, 2016, through April 30, 2021 (Figure 1). IN midazolam was administered in 214 (27%) of these visits. Patient demographics and clinical characteristics at the encounter level are presented in Table 1. Median ages were 4.99 (IQR 3.79-7.33) for the IN midazolam group and 5.15 (IQR 3.43-7.84) for the no midazolam group at the time of the ED encounter. IN midazolam administration was highest in preschool-aged children (3 to 6 years) (n=105, 50%) and non-Hispanic White children (31.7% vs. 19.3% Black and 6.1% Others). Prior bowel motility agent utilizations were 51.4% in the midazolam group and 34.4% in the no-midazolam group. Abdominal radiograph utilizations were 31.8% in the midazolam group and 28.9% in the no-midazolam group.

**Table 1:** Patient demographic and clinical characteristics at the encounter level

	No midazolam (n = 581)	Midazolam (n = 214)	Overall (N = 795)
Median age (years, IQR)	5.15 (3.43-7.84)	4.99 (3.79-7.33)	5.13 (3.52-7.71)
Toddler (<3 years)	102 (17.6%)	23 (10.7%)	125 (15.7%)
Pre-school (3 to <6 years)	236 (40.6%)	107 (50.0%)	343 (43.1%)
School-aged (≥6 years)	243 (41.8%)	84 (39.3%)	327 (41.1%)
Gender			
Female	254 (43.7%)	107 (50.0%)	361 (45.4%)
Male	327 (56.3%)	107 (50.0%)	434 (54.6%)
Race/ethnicity			
American Indian and Alaska Native	7 (1.2%)	4 (1.9%)	11 (1.4%)
Asian	61 (10.5%)	20 (9.3%)	81 (10.2%)
Hispanic	121 (20.8%)	42 (19.6%)	163 (20.5%)
Native Hawaiian and Other Pacific Islander	4 (0.7%)	3 (1.4%)	7 (0.9%)
Non-Hispanic Black	96 (16.5%)	23 (10.7%)	119 (15.0%)
Non-Hispanic White	215 (37.0%)	100 (46.7%)	315 (39.6%)
Other	77 (13.3%)	22 (10.3%)	99 (12.5%)
Prior bowel motility use	200 (34.4%)	110 (51.4%)	310 (39.0%)
X-ray imaging	168 (28.9%)	68 (31.8%)	236 (29.7%)

Primary and secondary outcomes are presented in Table 2. Overall, there was no statistically significant difference in the rates of successful BM between the IN midazolam (n=581) and no midazolam (n=214) groups. A total of 489 and 179 successful BMs were in IN midazolam and no midazolam groups, respectively (83.6% vs. 84.2%, p=0.94). When stratified by age in multivariate regression analysis, pre-school-aged children receiving IN midazolam had a slightly higher rate of success (92.0% vs. 83.0%, RR 1.09, 95% CI 1.01-1.18). There were no statistically significant differences in other age groups. Additionally, IN midazolam administration was associated with longer ED LOS (271 min vs. 235 min, difference 26.77, 95% CI 8.62-44.91), longer enema-to-discharge time (121 min vs 93 min, difference 19.49, 95% CI 8.48-30.5), higher frequency of multiple enemas (14% vs. 7%, p=0.021), and higher rate of admissions for constipation management (7% vs. 2%, p=0.002). There were no adverse events related to IN midazolam administration.

**Table 2:** Effects of midazolam based on regression analysis after adjusting for covariates for primary and secondary outcomes ^a^Risk ratios (RR) for binary outcomes and differences for continuous outcomes; modified Poisson regression analysis

Stratified analysis of successful bowel movement by age	No midazolam	Midazolam	Adj. RR (or difference^a^) (95% CI)	Adj. P-value
Toddler (<3 years)	94 (92%)	22 (96%)	1.05 (0.93 – 1.18)	0.469
Pre-school (3 to <6 years)	197 (83%)	98 (92%)	1.09 (1.01 – 1.18)	0.032
School-age (≥6 year)	198 (81%)	59 (70%)	0.87 (0.75 – 1)	0.055
Received >1 enema	41 (7%)	31 (14%)	1.77 (1.09 – 2.87)	0.021
Admissions	12 (2%)	15 (7%)	3.1 (1.5 – 6.41)	0.002
ED Length-of-Stay (min)	235	271	26.77 (8.62 – 44.91)^a^	0.004
Enema-to-discharge (min)	93	121	19.49 (8.48 – 30.5)^a^	<0.001

**Figure 2:**
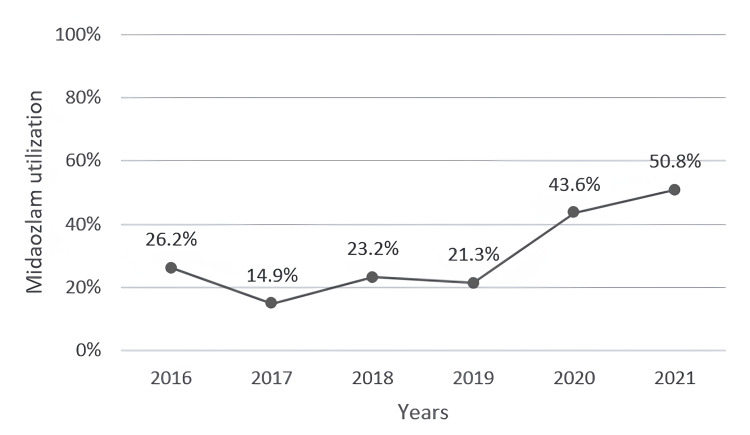
Midazolam utilization over the study period Single-center midazolam utilization for enema administration

IN midazolam utilization increased over the study period, with 50.77% of children being pre-medicated with IN midazolam prior to an enema in 2021 compared to 26.19% in 2016 (Figure 2). Bisacodyl was the most frequently administered enema (97.6%).

## Discussion

Overall, IN midazolam administration was not associated with a higher rate of successful BM after an enema. Rates of success were high in both groups, demonstrating the efficacy of an enema in producing stool. While preschool-aged children (3 to <6 years) receiving IN midazolam had a higher rate of success compared to those who did not receive midazolam, the effect was relatively small (RR 1.09, 95% CI 1.01-1.18). Children in this age group may have benefited more from anxiolysis due to their developmental age or other factors; however, given the small difference in relative risk, it does not appear to be clinically significant.

Contrary to our expectations, children who received IN midazolam had significantly worse secondary outcomes (ED LOS, enema-to-discharge time, multiple enemas, and hospital admissions). These findings may suggest a higher severity or perceived severity of constipation by the physician evaluating the child, as reflected by higher rates of prior bowel motility agent use and abdominal radiographs in the IN midazolam group. Another explanation is that anxiolysis and relaxation provided by midazolam had the opposite effect and impeded stooling by reducing discomfort and reducing the urge to defecate. The potential benefit of ED LOS may have been mitigated by the need for children to recover from sedation after receiving midazolam. Patient and family experience, an important impetus for providing anxiolysis in the ED, was unable to be assessed as it is not routinely documented in a quantifiable manner in our EMR. Similarly, other subjective but important metrics such as pain may have been affected by midazolam.

Midazolam utilization was higher in non-Hispanic White children. Prior studies have demonstrated racial and ethnic disparities in which non-Hispanic White children in pediatric EDs disproportionately receive more opioids for pain, undergo more chest radiographs for bronchiolitis, and receive more IV hydration for gastroenteritis compared to other groups [15-18]. Though disparities in care related to race are complex and the reason for the disparity in midazolam administration prior to enema is unclear, it is important to point out such differences when they are found and work toward standardization of care across race and ethnicity.

Consistent with current knowledge, IN midazolam was well tolerated without any adverse events. Increased utilization of IN midazolam during the study period was most likely reflective of our group’s increasing experience and comfort with using IN midazolam for procedural anxiolysis.

Limitations

First, as a retrospective cohort study, the quality of our data was dependent on the available records. With variable documentation of history and physical examination by different ED physicians and nurse practitioners, relevant clinical information may have been missed. For example, days without stooling, the presence of fecal impaction, and other findings that can help assess the severity of constipation were inconsistently documented and were not included in our analysis. Similarly, the decision to administer IN midazolam may have been influenced by these factors, leading to bias. Second, our study did not include children with neurodevelopmental disorders or prior abdominal surgery. Thus, it is not generalizable to this population. Whether children with sensory processing issues undergoing an enema will benefit from anxiolysis remains to be seen. Third, this study was performed at a single, freestanding children’s hospital ED and may not be generalizable to other institutions. However, neurotypical children with no prior abdominal surgery presenting with constipation to other EDs in North America are likely well represented by children in our study, given the high prevalence and ubiquity of childhood constipation. Finally, this study did not assess the patient anxiety and parent experience or prevalence of amnestic response related to IN midazolam.

## Conclusions

IN midazolam prior to the enema was not associated with an overall higher rate of successful BM in the ED. While there was a small improvement in stooling among preschool-aged children receiving midazolam, however, given the small difference, it is unlikely to be clinically significant. Although midazolam administration was associated with poorer secondary outcomes such as LOS, we suspect there were other confounding factors, such as the severity of constipation, that were unable to be assessed given the limitation of our study design. Future prospective studies are needed to evaluate midazolam’s potential benefit in preschool-aged children as well as its impact on subjective but important metrics such as patient and family satisfaction and anxiety.
